# Treatment Experiences Among Intimate Partner Violence Perpetrators: A Qualitative Analysis

**DOI:** 10.1177/0306624X221102846

**Published:** 2022-06-13

**Authors:** Moa Arvidsson, Shilan Caman

**Affiliations:** 1 Uppsala University, Sweden; 2 Karolinska Institutet, Solna, Sweden; 3 National Board of Forensic Medicine, Stockholm, Sweden

**Keywords:** intimate partner violence perpetrators, domestic abuse, group therapy, treatment needs, interpretative phenomenological analysis (IPA)

## Abstract

Intimate partner violence (IPV) is a global and widespread public health issue. Knowledge on what promotes individual-level reductions in risk for IPV recidivism is limited. In order to explore how group therapy influences the ability to obtain and sustain change, the objectives were to explore how IPV perpetrators make sense of and think about their experiences of group therapy and what their experiences are regarding needs of treatment for IPV perpetration. Six semi-structured interviews were conducted and analyzed with the qualitative method Interpretative Phenomenological Analysis. Two main themes were identified; “Experiences in group therapy” (subthemes: Ultimatum as a turning point; The guys as a powerful tool for change and Unfinished ending) and “Treatment needs” (subthemes: Violence as an addiction and Weakness as a strength). The findings highlight that group therapy is experienced positively, as well as difficulties that constitute obstacles, and need of further support after ending treatment.

Intimate partner violence (IPV) against women is a global public health issue, and the most common form of violence experienced by women. It has been estimated that approximately one in four women worldwide have been victims of IPV (World Health Organization [WHO], 2021), as such the WHO has repeatedly emphasized the need of actions to prevent violence against women (WHO, 2019). Risk factors for IPV can be found at multiple levels simultaneously: societal, community, relationship, and individual level, as such, interventions aiming to prevent IPV need to exist on multiple levels (Fulu & Miedema, 2015; Kouta et al., 2018). Individual-level risk factors; for example psychological or biological traits, psychological that influence the risk of becoming a perpetrator, is the focus of present study. Some suggest that IPV perpetrators have a greater extent of treatment needs compared to non-IPV violent perpetrators and nonviolent perpetrators. This highlights the importance of understanding the treatment needs in IPV perpetrators (Hilton & Radatz, 2018).

IPV often involves repeat offending and victimization (Frenzel, 2014). Therefore, effective treatment is a crucial step toward preventing recidivism in IPV perpetration, and of great importance from a victim safety perspective. However, systematic evaluations illustrate that there are too few studies of methodological rigor to determine whether the treatment interventions reduce violent behavior, and the evaluations to date demonstrate inconclusive and mixed results regarding the efficacy in current treatment programs (Eckhardt et al., 2013; Ferrer-Perez & Bosch-Fiol, 2018; Nesset et al., 2019). Heterogeneity among perpetrators of IPV may be one reason why individual studies show varying results. Subgroups within the population need to be distinguished, and treatment adapted thereafter, before clearer conclusions can be reached (Nesset et al., 2019). For example, one identified typology of IPV perpetrators is developed by Holtzworth-Munroe & Stuart (1994), where perpetrators of IPV were differentiated into subtypes based on the dimensions (1) severity and frequency of violence, (2) generality of violence, and (3) psychopathology.

There is limited qualitative research on how perpetrators of IPV have experienced treatment. The existing research has been summarized in a systematic synthesis of qualitative studies by McGinn et al. (2020), including both voluntary and court-mandated treatment. Common obstacles to change are cognitive distortions, emotional dysregulation, gendered social constructions, and self-esteem issues. Cognitive distortions are fixated ideas that support their problematic behavior, while emotional dysregulation consists of problems to understand and cope with emotions so that they are able to be non-violent. Gendered social constructions, such as patriarchal attitudes, is identified as obstacles to desist from violence. Furthermore, issues with self-esteem can make it difficult to seek help and show weakness to others, as their sense of self-worth is associated with displayed masculinity. The authors suggest that treatment should only be provided to appropriately motivated perpetrators, and that suitability of the group format should be considered in light of the heterogeneity of the population, as well as the complex nature of creating change.

Furthermore, the importance of the working alliance between the participants and the treatment provider is emphasized in treatment research (Santirso et al., 2020). Moreover, research on factors that influence the willingness to change highlights the importance of the treatment provider, but also underline the value of the other group participants for challenge and support (Gray et al., 2014; Holtrop et al., 2017; Morrison et al., 2019).

Scholars have encouraged future idiographic studies in order to examine whether IPV perpetrators perceive that support by the group contributes to desistance of violence (McGinn et al., 2020). Desistance denotes how people with a previous pattern of offending come to abstain from recidivating in crime. Walker et al. (2015) have developed a conceptual model of the process of desistance based on interviews with male IPV perpetrators (both court-mandated and self-referred), survivors and treatment providers. The model illustrates factors important for desistance from violence, such as external support and coping with triggers to violence. Morran (2013) conducted interviews with men who voluntarily sought treatment and were considered to have “successfully” completed treatment. Morran (2013) concludes that even committed men may require support over several years and that desistance from violence is better understood as a process rather than an outcome. Morran (2013) outlines three phases of desistance from IPV, *primary desistance* for when the person has ceased the pattern of violent behavior, in which techniques to identify and stop violence are of importance. In *secondary desistance* the person is continuously addressing behaviors and attitudes to reach a changed identity, where the termination of violence is more sustainable (Morran, 2013). Finally, *tertiary desistance* is where the changed person seeks new networks to support new priorities in life, and desires to give back to the community (Morran, 2018).

In order to gain increased clarity regarding what works for whom, the notion of heterogeneity among IPV perpetrators needs to be addressed, and the provided interventions adapted based on treatment needs. One step toward increased understanding regarding efficient treatment for IPV is to have more knowledge about the experiences of the individuals who themselves are targets of the intervention and who have completed such treatment. Particularly since dropout rates are relatively high (Askeland & Heir, 2013), it is beneficial to explore the provided treatment from their own perspectives.

In light of the inconclusive results from evaluation studies on treatment efficacy, and the scarcity of idiographic studies on experiences of these treatments, the present study aims to contribute to existing understanding on IPV by examination of individual accounts of how IPV perpetrators have experienced treatment. Knowledge of such may have relevance for development and adaptations of treatment interventions for IPV perpetrators. Thus, the purpose is to explore how therapeutic interventions and the format of the therapy has influenced IPV perpetrators’ ability to obtain and sustain change. The research questions are (1) how do men who voluntarily seek support for IPV perpetration make sense of and think about their experiences of group therapy and (2) what are their experiences regarding treatment needs for IPV perpetration.

## Method

### Design

The current study has examined IPV perpetrators’ experiences and treatment needs through semi-structured in-depth interviews, in which the recorded interviews were transcribed verbatim. The interviews were conducted and analyzed using a qualitative method called Interpretative Phenomenological Analysis (IPA). In accordance with IPA, the dialogs were not strictly guided by the interview guide, instead the interviews were characterized by enough flexibility to deepen the participants descriptions and to be able to follow up unexpected aspects during the conversation. IPA is particularly suitable when the study means to extensively analyze the personal experience and significance of the participants shared experience of a phenomenon (Smith, et al., 2009), and when the phenomenon in focus is a sensitive topic (treatment needs for IPV perpetration).

### Participants

The sample is part of a larger research project that has examined convicted and self-referred IPV perpetrators (*N* = *19*). This study has focused on the help-seeking individuals that have been recruited through a treatment center for men in crisis and with aggression issues in Stockholm, Sweden. A contact person at the treatment center assisted in identifying and asking potential participants who fulfilled the inclusion criteria. The criteria were: Male; minimum 18 years old; sought help for help/treatment for IPV; adequate skills of the Swedish language; ability to understand and give informed consent; and finished or about to finish group therapy. Violence was defined as physical violence (e.g., tearing, biting, hurting, pushing, slapping, or kicking), systematic threats about violence (e.g., threats to hurt the woman or mutual children), and sexual violence. Only IPV in heterosexual relationships were included. For a limited time, during February and March 2018, participants were continuously asked about participation in the study, and a movie ticket was provided (maximum value 100 SEK) as compensation for participation. In total, six individuals from the treatment center were interviewed, which was the number of individuals who met the criteria and accepted to participate within the time frame. The mean age of the participants were 39 years old (*SD* = 5.9 years). See table 1 for pseudonyms and sociodemographic information for each participant.

**Table 1.** Pseudonym and Socio-Demographic Information.

	Age group	Post-secondary education	Birth country	Relationship status
Erik	21–40	No	Sweden	Single
Amir	41–60	Unanswered	Unanswered	Unanswered
Per	21–40	No	Sweden	In a relationship
Aleksandr	21–40	Yes	Outside of Sweden	In a relationship
Johan	41–60	Yes	Sweden	In a relationship
Niklas	41–60	No	Sweden	In a relationship

The group therapy provided at the treatment center is mainly influenced by psychodynamic therapy, but with influences of cognitive/behavioral, psychoanalytical, and psycho-educative methods, and is led by licensed psychologists or psychotherapists. It consists of 15 sessions; however, participants may attend further sessions if needed. Each session starts with the question “how has your week been?” in order to raise themes that are current. This is believed to increase engagement and willingness to take responsibility for own change.

### Procedure

The participants were asked about study participation in the end of, or after completed, treatment by a contact person, in an orally neutral way with the assistance of a template. The participants were also informed more extensively about the study in a written form, which among other things included information about the right to withdraw at any time without the need to provide an explanation. After the written consent was obtained, the recorded interviews were conducted one-on-one by the second author SC at the treatment center. The interviews revolved around primarily two areas: Experiences of perpetrating violence and experiences and needs related to interventions for IPV, in which the focus of present study is on the second area of questions. The interviews, that lasted on average 90 minutes, were thereafter transcribed verbatim. The participants also filled out a form with questions about sociodemographic background and criminal history. The study was approved by the Regional Ethical Committee in Stockholm, Sweden (Protocol ID 2017/2371-31/5).

### Analysis

The initial step in analyzing the interviews was to listen to the recordings, thereafter the transcriptions were read several times while associations and comments were documented line by line (Smith et al., 2009). Prominent themes were identified in the next step and labeled for each individual interview, and connections between these themes were analyzed. Cluster of themes were comprehended to more overarching themes that were further analyzed. Thereafter, patterns between each interview were analyzed and the transcriptions were interpreted on different levels, both as a whole and in detail, to capture the depth of the descriptions.

## Results

The analysis of the interviews resulted in two overarching themes: *Experiences in the group therapy* and *Treatment needs*. As presented in Table 2, each overarching theme consisted of subthemes.

**Table 2.** Identified Themes and Subthemes, Including Examples of Where in the Transcript (Page Number: Line Number) Interpretation for Each Theme is Found for Every Participant.

	Aleksandr	Amir	Erik	Johan	Niklas	Per
Experiences in the group therapy						
Ultimatum as a turning point	8:39	12:19	11:6	16:33	16:43	12:15
The guys as a powerful tool	14:28	11:1	14:47	16:12	23:6	14:9
Unfinished ending	11:13	12:28	17:19	18:24	17:23	17:50
Treatment needs						
Violence as a substance addiction	11:29	11:1	–	–	12:45	–
Weakness as a strength	10:45	13:37	16:23	17:22	18:47	16:1

### Ultimatum as a Turning Point

This theme captured the participants’ experience in the beginning of the therapy of how difficult it was to seek help but despite the resistance lead to a sense of relief. As such, the ultimatum became a turning point where they not only felt external motivation from the pressure of their surroundings to seek help, but also over time described internal motivation to change their own behavior in order to make themselves feel better. The theme was primarily evident by participants’ descriptions about needing external pressure from their surroundings in order to seek help, either from a spouse, a friend, or even in the form of a police report. The turning point was not necessarily reached prior to treatment, rather the treatment itself was often seen as a contributing factor to realizations that motivated a desire to change. Calling the treatment center was experienced as a much bigger step than merely dialing a phone number, as they did not know what to expect. Their emotional experiences seemed to shift as they decided to seek help.

Per: So there was some kind of relief, that now I am really going to deal with this, because damn it, I am going to become. . . but I guess that was also the first time that I confessed to myself that I. . . fuck you really need help [. . .].

This extract demonstrated how Per, despite the struggle to seek help, rather experienced a newfound strength, which his use of “damn” further accentuated. Per described how he had avoided admitting he needed help, which may mean he expected it to have a negative impact, when the ultimatum on the contrary led to a sense of relief. The discrepancy between expectations and the real-life consequences may therefore be a part of the explanation of why they had struggled to seek help without some sort of external pressure. As time went on, the motivation for going to treatment seemed to shift as well.

Amir: Before when I started coming here it was only a chance to talk and get tools to make her understand that it is wrong with her [laughing] and not me. But then when one got here and listened and listened it was just like, wait a minute. Inspect yourself and change yourself first, before you change others.

Amir illustrated that the therapy changed his perception of what was the problem in his relationship. Even though he lacked desire to change his own behavior prior to entering treatment, as it progressed, he gained insights that made him want to change. According to the participants’ descriptions the ultimatum was a driving force for external motivation to change, which caused them to seek help. However, over time their internal motivation to change for themselves increased. Several participants also reasoned about how a police report was a compelling and necessary force to change their behavior, without being directly asked. Despite its’ potential to ruin their current family life, a police report was in retrospect described as a savior. It seemed to signify their conflict between wanting to avoid their problems, and a yearning for boundaries forcing them to face them. In conclusion, this theme demonstrated how the experience of an ultimatum was complex. It represented a sort of culmination of their problems and was a difficult time. On the other hand, it was a symbol for a turning point that gave them a sense of relief, as they dared to take the step and seek help.

### The Guys as a Powerful Tool

This theme illustrated how the other participants in the group therapy consistently were described as the tool that affected them the most. When they were asked whether anything was helpful in the group therapy, they often mentioned stop techniques in potentially violent situations as valuable, although they particularly emphasized the value of the other participants. It was also mentioned spontaneously in the interview, which further showed how prominent the theme was. The “guys” were described as a tool that made them more susceptible to change and that gave support in the difficult process. Listening to other participants’ stories led to some kind of automatic comparison to themselves, which was described as an efficient method to realize how problematic and skewed their usual justifications for violent behavior was. It also was described as a motivating factor, either by striving toward, or being deterred from, becoming as other participants. As other group participants opened up, they received a natural urge to reciprocate and to also share.

Aleksandr: I did not like to seek support here, I did not like that I would have to share in front of others. It felt incredibly weird. But then, when I arrived here the first time, I realized that they really have the same issues. And all of a sudden it felt pretty safe. It immediately felt safe. It felt a bit like. . . damn, I can really tell here. If to anyone, I can [speak] to these guys.

The extract above showed what a significant impact the other participants had on the experience of the group therapy. Having realized that the other participants shared the same difficulties, Aleksandr’s experience shifted from unsafe to safe. It showed that his reluctance to open up didn’t stem from not wanting to, but rather from a fear of people not understanding. His emphasis on “if to *anyone*” illuminated his longing for finding a place where he finally felt safe enough to share his experiences and needs. The participants also described a desire to be called out when they lied, which felt easier to get from the other participants than the therapists, since they had a more direct approach and a genuine understanding.

Amir: [. . .] And then I said that ’here you are not being honest, here you are lying, so on and so on and so on and so on’ and then that person got frustrated, because he knows I’m right. So that’s how I learned from everything, when I listened and like. . . gave advice.

It seemed in Amir’s reasoning as if he was trying to provide with what he desired himself, namely help to be fully honest. They did not describe that the role obscured their own needs in treatment, rather learning in the group was achieved by both getting feedback and giving it. Despite awareness that honesty was key to improvement, they described difficulties being honest on their own, and the other participants therefore became a helpful tool to be more honest. The “guys’” importance was emphasized to the extent it seemed they were considered more legitimate as therapists. The therapists’ role to structure and lead the group therapy was seen as important, however, as each participant came further along in the change process the therapists’ significance diminished, but not the other participants. The diminished need of therapists can be understood as an increased ability to manage problems independently, while the role of the other participants continued to fill a need for sharing. Other than having an ability to give good advice, the participants were described as important for additional reasons.

Niklas: I gain huge trust for such a person. He beat up his wife, and was fired and went to prison, but now he doesn’t [perpetrate violence]. Now he is the opposite, he is warm and never bad to anyone. That means he must have done something, then I want to do that as well. /. . ./ That is what you want, that is attractive. It is so shameful and embarrassing and difficult. And it should be, but at the same time, it makes it harder to change.

The quote above illustrated that the importance of the other participants was not necessarily due to their actions in the group, it was rather the hope and trust that resonated with Niklas. It was as if they felt safe to not be judged by the other participants. While therapists on the contrary may have been seen as having the upper hand as they did not share personal issues. As a consequence, the fear of being judged was not as easily removed. Niklas described an inner struggle where he wanted to change but had difficulties sustaining the motivation if he did not concretely see how that effort led to his increased well-being. Niklas’s feelings of shame seemed to be experienced as an obstacle to change, which the other participants helped with. The feelings the participants had when making a change therefore seemed crucial for motivation, as increasing shame lessened the desire to proceed. While the other participants served as a sort of living example of the positive aspects of the change that also they could reach created a desire within the individual to continue. In conclusion, it was prominent what an immense impact the other participants had. They described an inner struggle during the group therapy, and the group provided essential support in order to cope with the struggle.

### Unfinished Ending

This theme illustrated the experience of ending the group therapy, which was described as the opposite of an actual ending, that is being unfinished. It was primarily manifested in that all participants wanted continued support in different ways, and that most of the study participants have sought some kind of healthcare after completed treatment. They portrayed that changing was a lifelong process and requested support to gradually manage on their own. Despite the participants not being asked directly how they felt about ending treatment, some participants described a sense of abandonment.

Johan: It is one thing that we are going to prevent intimate partner violence and then try to find a way to do so, but for those who have made that journey, I think you would feel good if. . . like, step two in this course is how do you build a healthy and good relationship? With the baggage that you have? [. . .] well maybe that’s what you’re expected to manage on your own. That makes me think that the society is more concerned with like ‘okay as long as he stops using violence and being a burden to society, we are happy, and what happens to him thereafter is his own concern.’

A feeling of abandonment developed as Johan described how he felt an expectation to manage on his own after he stopped perpetrating violence. His reference to society seemed to illustrate that he did not feel abandoned by a particular person but by society at large. It may have shown his desire to feel like an asset instead of a burden to society, but he felt the need of continued and deepened support to reach further desistance. He resembled his experience of the group therapy with a journey, where his needs regarding how to desist from violence (i.e., primary desistance) had been met but not the next step regarding how to build healthy relationships. The participants described the end of the group therapy as a sort of shift in their experience, where they both had accomplished great change and realized they wanted to change far more than they had. As such, the participants requested a continuation of the therapy, through individual therapy, couples therapy, men’s groups, and mentoring by previous IPV perpetrators who had “successfully” changed. Other than a support system in times of need, mentors have been described as a long-term motivation to sustain their own change and an opportunity to give back to society in the future.

Aleksandr: I can’t demand it from my friends, my buddies, to always like, you know. . . In most cases, it was unfortunately like, damaged family relationships, some missing parent, somebody that had left, somebody that you just have a totally fucked up relationship with. So, there is this, I guess there is this need for validation, to get a bit of energy somewhere.

The quote above highlighted how mentors represented a complex need that seemed to stem from unmet needs in their existing relationships. Aleksandr’s experienced that he cannot demand that kind of support from his friends, which may both have reflected a realistic observation that friends cannot fill the same function as the group and his expectation that his needs would not be met if he tried. That it would also fulfill a need for validation meant mentors may be connected with their difficulties and attempt to strengthen a low self-esteem. A long-term goal would therefore be to find validation within themselves, rather than through others. While after the end of treatment mentors remained important to give them support to sustain change and desistance from violence. The participants also described the ending as unfinished by describing time as a key factor to accomplishing change.

Niklas: It is a bit too little. I don’t think you solve a 25- to 30-year-old problem with violence in five or ten times. I think it takes many years to work with.

Niklas described that no matter how the group therapy is outlined, the necessity of time could not be avoided. The ending is therefore inevitably unfinished in some way. However, the theme highlighted a mismatch between the number of sessions of the therapy and the participants experience of their need of support in a lifelong process, where primary desistance merely is the start. In conclusion, the ending of the group therapy was experienced as difficult, and the participants seemed to come to terms with never really becoming “finished.” However, instead of an abrupt ending they seemed to need a gradual transition into managing without the crutch the group therapy came to be. Their request of mentors showed that support should be based on current needs and strive to help them build strength within themselves.

### Violence as a Substance Addiction

This theme captures the respondent’s tendency of comparing and associating violence with a substance addiction, which partly illustrated their needs of help in order to sustain desistance from violence. Even though the theme was only expressed by some participants and on few occasions, it was a theme that was primarily illustrated in the participants’ language. By for example using the word relapse to describe whether they had, or risked to, perpetrate violence again. The continued need of help in order to sustain change, which the group therapy had begun, was however described by all participants by emphasizing the constant struggle they experienced with changing their habits and maintaining new ones. The participants who also used the resemblance to substance addiction seemed to use it as a way to explain shifting between feeling empowered and helpless to sustain a lasting change.

Aleksandr: Alcohol abuse, for example. You take the first beer, and you are screwed. The same with violence. If you allow yourself again, then it is easier to end up there. So, it is unfortunately a constant struggle.

For Aleksandr the constant struggle seemed to be what made his experience of desisting from violence feel like a substance addiction. Through saying “if you allow yourself again” he showed the conflict-ridden choice he continuously faced. When he described how it is enough with a single beer before it is too late, he showed how the choice he was struggling with was not necessarily to stop his violent behavior, but to prevent taking any steps that could lead to the pathway to violence. Helping the participants to sustain the change is therefore complex and entails helping them understand the signals that precede a violent situation and supporting them in making the decision to stop as early as possible. Based on their descriptions, the participants seemed to have focused on what they wanted to avoid. Thus, in order to facilitate their own motivation of sustaining change they also seemed to need help with making goals of what they wanted to strive toward. The metaphor of violence as a substance addiction was not only important to describe their experience of the struggle but also to describe if they had, or risked to, perpetrate violence again.

Niklas: Then I stopped here, and one is a bit inattentive with the other parts [violent behaviors] and working, and then it came back again at some point. It didn’t turn out quite well, and every time it was like a relapse, as if I took a relapse in drugs.

The quote above illustrated how the word relapse was an important word in the participants’ vocabulary. It may have been close at hand because a few of the participants had personal experiences of substance addiction. The metaphor of violence as a substance addiction may therefore partly reflect a common comorbidity. It also seemed to be a way to explain their attempts to be non-violent by using the word relapse. In conclusion, violence as a substance addiction was a metaphor that described how the participants experienced desisting from violence as a constant struggle because changing habits seemed to create an inner conflict. The present theme illustrated treatment needs to feel empowered to sustain the change and to have goals to strive toward.

### Weakness as a Strength

This theme addressed the participants need to see it as a strength rather than a weakness to express emotions and show their most vulnerable sides in the group therapy, and especially outside of it. The theme was apparent for most participants by expressing thoughts about how they were perceived by others in the group therapy, and that they felt an instinct to adjust their behavior so that they would be presented in a desirable way. The desire to be liked could even be so strong that they sometimes did not share reoffences in IPV, which as a consequence made it harder to receive proper help with their struggles. However, the group therapy was often described as a safe space where they were able to show more vulnerable sides, while they had more difficulties finding places outside of the group context in order to vent. One factor that several participants described made it difficult to show vulnerability was a fear to appear weak. This following quote illustrated how the need to release painful feelings conflicted with a need to appear strong, which was brought up in a context where the participant shared difficult events during his upbringing in the group therapy.

Aleksandr: [. . .] there is a certain need to, hide certain things, to not be judged by people who haven’t experienced what I have, because of these situations. There is a need to appear as, I don’t know, strong if that is the right word. But there is a need in me, and then revealing the hard parts, that’s not particularly fun.

The quote illustrated the resistance Aleksandr experienced related to sharing difficult events in the group therapy. It seemed as if the participants struggled to talk about these events not only due to the emotions they brought, but also because showing these painful emotions was associated with tearing down the strength they liked to demonstrate. The participants described needing support in order to both endure painful emotions and to realize that showing weakness is courageous. A fear of other people’s reactions was also described as a reason why it could feel so difficult to show vulnerability.

Erik: [. . .] this with expressing myself has built up so much in my head . . . that it might be these [big] consequences, because of something I say, something that I think, when there doesn’t need to be any consequences at all or . . . judging at all or so. More understanding.

In the quote Erik showed how his focus on the potential disastrous consequences of what might happen if he expressed himself became such an obstacle that even speaking his opinion was difficult. Through the group therapy Erik seemed to have realized that his fears were not realistic, when he tested expressing himself in the group therapy he was instead met with understanding. The group therapy therefore served as a sort of correctional experience where the participants could test the legitimacy of fears related to expressing themselves.

A participant, Amir, described the group therapy as a venting space where he got energy from talking about what is good, and also got to drain the bad things that needed to get out. However, despite realizing how good it felt to share in the group therapy, the participants seemed to have struggled more to find places to express themselves in everyday contexts outside of the group therapy. Perhaps the group therapy felt like an exception rather than a rule that they would be met with a positive reaction if they expressed themselves. Therefore, the participants described a need to express themselves, both in the group therapy and particularly to continue to do so in their everyday lives, so that they don’t necessarily need to wait until their group therapy session to finally vent. Amir further explored why it was important to express emotions during the interview.

Amir: [. . . ] when it was the most difficult nobody, even though school and teachers and everybody knew there were problems and such at home, nobody sat with you and said ‘it’s okay to talk, it’s okay to cry, it’s okay to show emotions’. So, I never got to [talk], except for when I was alone or angry, so it’s hard for people to know.

Interviewer: How do you think that affected you? I mean that you didn’t get a chance to express emotions and know it’s okay?

Amir: Well, it affects you in the way that you tend to hold things in, and it explodes eventually, everyone needs to talk, and I have noticed that.

In his description Amir showed how he experienced it unacceptable to express emotions unless he was angry or alone, which caused the emotions to eventually explode. In order to avoid recidivism in violent behavior in the long-term, the participants seemed to have a great need to increase the perceived freedom to express their emotions and learn how to avoid building up emotions until they are overwhelmed. Meanwhile, stopping techniques to calm down in potentially violent situations were important short-term strategies. This entails a paradox to some degree; they had a need to control their emotions and learn to express them freely at the same time. By Amir saying that he had noticed that everybody needs to talk may have meant that the group therapy helped him realize his need of expressing himself.

In conclusion, the participants descriptions demonstrated both a strong desire and fear to show vulnerability and express emotions. Fears about negative reactions from others and a belief of needing to appear strong prevented them from truthfully expressing themselves, although the group therapy was experienced as a safe place to vent. One can argue they needed support to see it as a strength rather than a weakness to show vulnerability since it meant having the courage to face fears. By allowing themselves to open up they could find new places to vent, even after the ending of the group therapy.

## Discussion

The overall objective was to explore how therapeutic interventions and the format of the therapy influence IPV perpetrators’ ability to obtain and sustain change. More specifically, we explored how men who voluntarily seek support for IPV perpetration make sense of and think about their experiences of group therapy. The results demonstrate that the group therapy is experienced as a complex, yet fruitful, experience. Overall, it was described as difficult because it meant addressing issues they had avoided for a long time, positive since they felt a great support, and above all important because they perceived it started a change to desist from violence. The results indicate several important areas that therapies targeting IPV perpetrators should contain in order to induce a positive and lasting change. Obstacles they needed help with to address were difficulty being vulnerable and fear of reactions from the other group participants; to stay motivated while struggling with change; and to sustain the positive change over time.

In line with previous research (Chovanec, 2014; Gray et al., 2014; Holtrop et al., 2017; Morrison et al., 2019; Walker et al., 2015), the other participants in the group therapy are perceived as valuable to obtain change. Our study further demonstrates that further along in the therapy, the perceived value of other participants seemed to exceed the value of the therapists, which highlights the perceived positive influence of other participants. McGinn et al. (2020) questioned whether the group format actually influenced desistance from violence other than being experienced as positive. Findings from our study demonstrate that the group format is experienced as helpful in terms of support and motivation to change, being more honest in the group and increasing openness in sharing difficulties. In other words, the group format may indeed contribute to desistance. However, it is worth pointing out the differences in samples, the participants in our study sought treatment voluntarily while court-mandated IPV perpetrators were included in the study by McGinn et al. (2020). Group therapy in a prison environment may on the contrary have an inhibitory effect if the environment is experienced as unsafe to share personal difficulties.

The decision to be non-violent has to be made by the individuals themselves. However, as demonstrated in the theme “ultimatum as a turning point” and previous studies (Gottzén, 2019; Walker et al., 2017), external negative events, such as possible criminal sanctions or being left by a partner, are perceived as motivational factors by IPV perpetrators. Some described external pressure from an ultimatum as necessary in order to accumulate enough motivation to seek help, in which the external motivation over time was complemented by internal motivation to change for themselves. However, it has been suggested that treatment only should be provided to appropriately motivated IPV perpetrators (McGinn et al., 2020). However, our findings indicate only few IPV perpetrators would be eligible for treatment, if the requirement for would be based on motivation. Instead, the focus should be to increase internal motivation as the treatment progresses. Based on the IPV perpetrators’ descriptions, such a change may be a natural part of the overall change process. Therefore, rather than considering a crisis to be an obstacle, it may be symbolized as an opportunity.

Our findings, especially highlighted in the theme “weakness as strength,” indicate that one important aspect in order to sustain a long-term desistance from violence is the ability to express emotions. Emotional dysregulation has been identified as a target for treatment in IPV perpetrators (McGinn et al., 2020). Also, emotional dysregulation mediates the relationship between restrictive emotionality and aggression, in which it can be primarily driven by emotional dysregulation in the form of lack of acceptance and tolerance of emotional experiences rather than the inability to regulate internal experiences. The propensity to use violent behaviors may increase if these are considered to be the only acceptable forms of emotional displays (Cohn et al., 2010). Therefore, expressing emotional experiences in a healthy way may be key to sustaining non-violent behaviors. On a similar note, our findings suggest that shame is perceived as an obstacle to be receptive for treatment and to change. Acknowledgement of shame, for perpetrators who experience feelings of shame, may be a factor that separates “desisters” from “persisters” of violence (Walker et al., 2017). Moreover, the experience of professionals working with IPV perpetrators is that hypermasculine attitudes contribute to difficulties expressing emotions and vulnerability in IPV perpetrators (Morrison et al., 2021).

The findings in theme “violence as a substance addiction” were novel, in which perpetrators use substance addiction to describe their experiences of IPV perpetration and struggles with desisting from violence. The theme “unfinished ending” further illustrated that the participants had a need of support to continue the change that the group therapy started. That desistance of violence is best understood as a process is supported by Morran (2013) and Walker et al. (2015). The latter also illustrate in their model that IPV perpetrators can resume violent behavior after new lifestyle behaviors have been created, which can be resembled to how the participants in this study experienced violence as a substance addiction. However, they also described time as an important factor in itself for making a change and therefore the process of changing may not be something to complete. That most participants in this study sought treatment again after it was finished illustrates that after learning to desist from violence, they may need a step two in the treatment based on their individual needs, such as how to build healthy relationships. A question is where to set the limit with regards to resources for provided IPV treatment. If the limit is set for primary desistance of violence, the participants only receive help in the first step of a long changing process, which may not be sufficient for preventing recidivism in IPV.

### Limitations

The present study is not without limitations. Firstly, the interviews rely on the respondents’ memories, as they are asked to recall how the group therapy has been in the past. As such, limitations associated with human memory apply. Secondly, their descriptions may build on treatment narratives and what they have learned during the therapy, rather than their own experiences and reflections. Thus, the results that builds on their perspectives have in turn been influenced by the group therapy. Furthermore, as the participants were asked sensitive questions and about a topic considered taboo, there is a risk they may have provided responses adjusted to be presented in a more socially desirable way.

### Implications for Research and Practice

Given that ultimatums and negative events have been described as a turning point, treatment needs to be available during crises so that important windows of opportunity are seized while external motivation is present. However, this may also indicate that internal motivation may be low at the start of the treatment. Thus, interventions targeting motivation, such as motivational interviewing, may be of particular importance in IPV treatment and can reduce dropout and recidivism (Santirso et al., 2020). For instance, police officers who come in contact with individuals in crisis and potential perpetrators of IPV can strategically provide information to relevant authorities and encourage them to seek help. In order to prevent recidivism in IPV, it is of great importance to have low thresholds to initiate contact with a support or treatment provider.

There was perceived to be many positive aspects with therapy in group, therefore the group format has many practical advantages compared to individual therapy for IPV perpetrators who voluntarily seek treatment. Future research may benefit from distinguishing individuals who are court-mandated and who voluntarily seek treatment in this regard. In addition, increased acknowledgement of typologies of IPV and IPV perpetrators may provide more nuanced information on what works for whom, and under what conditions (Armenti & Babcock, 2016; Nesset et al., 2019). Even though the participants experienced the group therapy as an important source of support, our findings also illustrate that it was not enough to further the process of desistance past a primary phase. Therefore, one important aspect to consider is whether continued support after completed group therapy should be offered. Availability of further support and treatment, based on individual needs, could be a necessary step to support the process of further and deepened desistance (Shamai & Buchbinder, 2010). Also, networks of mentors, consisting of previous IPV perpetrators, could be a contact to reach out to in times of need of support, and thus play a crucial role in preventing recidivism.

Our study indicates that IPA as a method is an efficient method and approach for interviews involving perpetrators of IPV. There was room in the interviews for developing rich and nuanced data that focused on allowing the participants to elaborate and in detail describe past and present experiences. It also made it possible to build an alliance, which contributed to the participants expressing themselves more freely in the interviews.

## Conclusions

Our study highlights several areas that may be important for informing and adapting support and treatment interventions targeting IPV perpetrators. For instance, difficulties feeling shame and vulnerability, and inability to express emotional experiences were described as obstacles. In light of ultimatums and negative events being described as turning points, treatment ought to be available during crises, in order to seize the windows of opportunity for intervention. Group therapy was described as a complex, yet fruitful, experience: difficult because it meant addressing issues they had avoided for a long time, positive since they felt a great support, and as the start of a changing process to desist from violence. However, availability of further support and treatment, based on individual needs, could be a necessary step to support the process of desistance past a primary phase.
